# Single Nucleotide Polymorphisms of the Sirtuin 1 (SIRT1) Gene are Associated With age-Related Macular Degeneration in Chinese Han Individuals: A Case–Control Pilot Study

**DOI:** 10.1097/MD.0000000000002238

**Published:** 2015-12-11

**Authors:** Zhiqing Chen, Yi Zhai, Wei Zhang, Yan Teng, Ke Yao

**Affiliations:** From the Eye Center, Second Affiliated Hospital of Medical College, Zhejiang University, Hangzhou, Zhejiang, China (ZC, YZ, YT, KY); Key Laboratory of Ophthalmology of Zhejiang Province, Hangzhou, China (ZC, YZ, YT, KY); and Department of Pathology, Zhejiang University School of Medicine, Hangzhou, China (WZ).

## Abstract

To investigate whether 3 variants in sirtuin 1 (SIRT1) gene contributed differently in patients with age-related macular degeneration (AMD) in a Chinese Han population.

We conducted a case–control study in a group of Chinese patients with AMD (n = 253) and contrasted the results against a control group (n = 292). Three single nucleotide polymorphisms (SNPs) of SIRT1 gene including rs12778366, rs3740051, and rs4746720 were genotyped using improved multiplex ligase detection reaction. The association between targeted SNPs and AMD was then analyzed by codominant, dominant, recessive, and allelic models.

The genotyping data of rs12778366, rs3740051, and rs4746720 revealed significant deviations from Hardy–Weinberg equilibrium tests in the AMD group but not in the control group.

We detected significantly differences of rs12778366 allele distribution between 2 groups in recessive and codominant model (*P* < 0.05). Homozygous carriers of the risk allele C displayed a higher chance of developing AMD (*P* = 0.036, odds ratio = 3.227; 95% confidence interval: 1.015–10.265).

Our study, for the first time, raises the possibility that genetic variations of SIRT1 could be implicated in the pathophysiology of AMD in the Chinese Han population.

## INTRODUCTION

Age-related macular degeneration (AMD) is 1 of the leading causes of severe visual loss in developed countries^1,2^ and in China.^3^ Late AMD can be divided into 2 subtypes: geographic atrophy (dry AMD) and neovascular AMD (wet AMD). Some scientists suggested that frequency of AMD subtypes in Asians may be different from Caucasians.^4,5^ In Asian populations, neovascular AMD is the major subtype of late AMD and is featured with choroidal neovascular membrane (CNV).^6^ The pathologic hallmark of this disease is drusen, deposits of proteins and lipids, in the Bruch's membrane; these deposits, along with pigmentary irregularities, constitute early AMD.^7^ Though, several genes have been reported to be associated with this disease,^6,8–10^ the pathophysiology of AMD is still not well understood yet.

The sirtuins (SIRT) are a highly conserved family of NAD-dependent class III deacetylases that helps to regulate the lifespan of diverse organisms. Mammalian sirtuins consist of 7 members, SIRT1–SIRT7, and some of them, especially SIRT1, have been shown to play crucial roles in the regulation of aging, longevity, or in the pathogenesis of age-related metabolic diseases.^11,12^ In human eyes, SIRT1 expression has also been observed in the lens epithelium of patients with age-related cataract,^13^ adult retinas,^14^ and corneal epithelium.^15^ SIRT1 protects the retinal cells from oxidative stress-related retinal damage, apoptotic retinal death, and anti-inflammation.^16^ Bhattacharya et al^17^ showed that P53 acetylation Lys379 in primary human retinal pigment epithelium (RPE) increased though the SIRT1 inhibition, and hypothesized that treatment target inhibition of p53 phophorylation or acetylation may prevent RPE cells from apoptosis. Recently, Maloney et al examined SIRT1 expression in excised human choroidal neovascularization membranes and non-AMD donor eyes by immunohistochemistry. They found SIRT1 levels elevated in human choroidal neovascularization membranes compared with control eyes.^18^

These observations suggest the possibility that SIRT1 is a candidate for conferring susceptibility to AMD. In order to test this hypothesis, we investigated the association between the single nucleotide polymorphisms (SNPs) within the SIRT1 gene and AMD in Chinese subjects. Three well-studied SNPs were analyzed: rs12778366^19–21^ and rs3740051^22,23^ in the 5′-flanking region of the SIRT1 gene and rs4746720^24,25^ in the 3′-untranslated region.

## MATERIALS AND METHODS

### Patients Recruitment

The protocol adhered to the tenets of the Declaration of Helsinki. This study was approved by the Ethics Committee of Zhejiang University. Written informed consents were obtained from all subjects. This case–control study included 253 unrelated Chinese patients with AMD in at least 1 eye and 292 unrelated control subjects recruited in the Second Affiliated Hospital of Medical College, Zhejiang University, Hangzhou, China from 2011 to 2014. The diagnosis of AMD was confirmed in case group by ophthalmoscope examination, color fundus photography, ﬂuorescein and indocyanine green angiography, and optical coherence tomography. Patients having any of the following characteristics were excluded from the study: high myopia (spherical equivalent > 6.00 diopters); macular atrophy caused by other reason such as trauma, inflammation, and vascular disease; other neovascularized maculopathies such as angioid streaks, retinal angiomatous, proliferation; and polypoidal choroidal vasculopathy. The control subjects were recruited among patients who underwent routine health examinations, and willing to participate in the study. All control subjects were aged ≥45 years. They all had visual acuity measurements, slit-lamp biomicroscopy, ophthalmoscope examination, and color fundus photography.

### Genotyping

DNA was isolated from peripheral blood mononuclear cell of the recipients using the QIAamp DNA blood mini kit (Qiagen, Inc., Hilden, Germany). The SNP genotyping was performed using a patented improved multiplex ligation detection reaction technique (iMLDR, Genesky Bio-Tech Cod., Ltd., Shanghai, China). The primer and probe information in 2 mixtures are described in Tables 1 and 2, respectively. The multiplex polymerase chain reaction (PCR) was carried out on the ABI Veriti thermal cycler (Applied Biosystems) in a total volume of 10 μL, including 1 μL genomic DNA (contain 5–10 ng DNA), 1 μL 1× GC-I buffer (Takara, China), 3.0 mM Mg^2+^ (Takara), 0.3 mM dNTPs, 1 μL primer mix (1 μM each primer), and 1 U HotStarTaq polymerase (Qiagen Inc.). Cycling parameters were as follows: 95°C for 2 min; 11 cycles at 94°C for 20 s, 65°C and −0.5°C per cycle for 40 s, 72°C for 1.5 min; 24 cycles at 94°C for 20 s, 59°C for 30 s, 72°C for 1.5 min; then 4°C forever. Thereafter, 5U shrimp alkaline phosphatase (SAP) and 2U Exonuclease I (EXOI) were added to 10 μL of PCR product for purification. The mixture was incubated at 37°C for 60 min, followed by incubation at 75°C for 15 min. Then, 2 μL purified PCR product was mixed with 1 μL 10× ligase buffer, 0.25 μL ligase, 0.4 μL 5′ ligation probe mixture (1 μM), and 0.4 μL 3′ ligation probe mixture (2 μM), and 6 μL ultrapure water for ligation reaction. The ligase detection reaction (LDR) parameters were as follows: 38 cycles at 94°C for 1 min and 56°C for 4 min, then 4°C forever. Following the LDR reaction, 0.5 μL LDR reaction product was mixed with 0.5 μL Liz 500 size standard and 9 μL Hi-Di, and incubated at 95°C for 5 min before loaded onto an ABI3730XL Genetic Analyzer (ABI). Data were analyzed using GeneMapper software (version 4.1, Applied Biosystems).

**TABLE 1:**
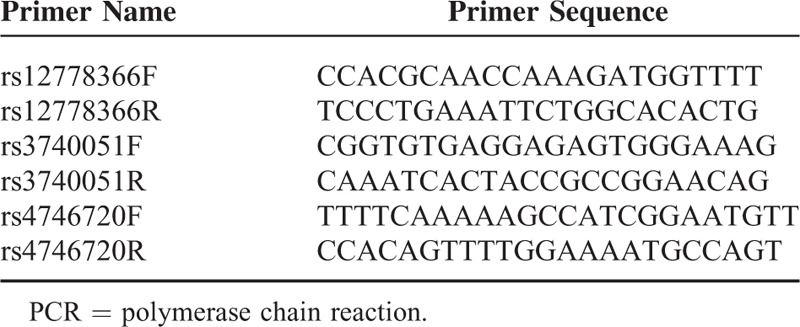
Primer Sequence and Concentration in PCR Mixture

**TABLE 2:**
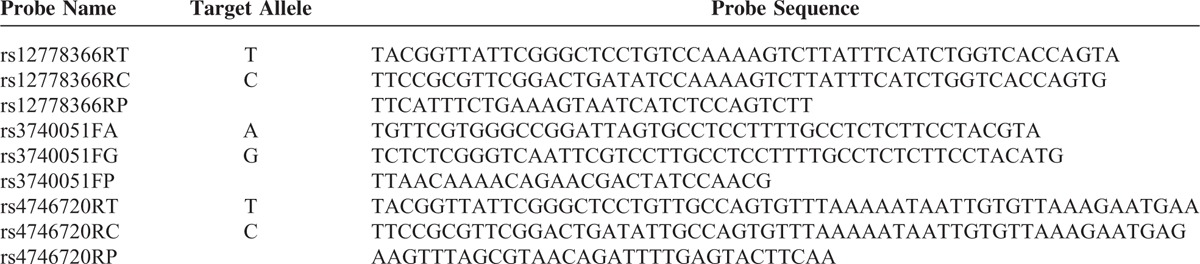
Probe Sequence and Concentration in Probe Mixture

### Statistical Analysis

All SNPs were evaluated for Hardy–Weinberg equilibrium (HWE) using the χ^2^ test (1 degree of freedom) with STATA software, Version12.0 (StataCorp LP, College Station, TX). The χ^2^ test was used to compare categorical allelic and genotype distributions between cases and controls. The odds ratio (OR) and corresponding 95% confidence interval (CI) were calculated relative to the major allele and the wild type homozygote. Analyses were performed with the SPSS 13.0 for Windows (SPSS Inc., Chicago, IL). Significance levels were set at *P* < 0.05.

## RESULTS

A total of 545 subjects were enrolled in this study, including 253 patients with AMD and 292 control individuals. Demographic information about the cases and controls are listed in Table 3.

**TABLE 3:**
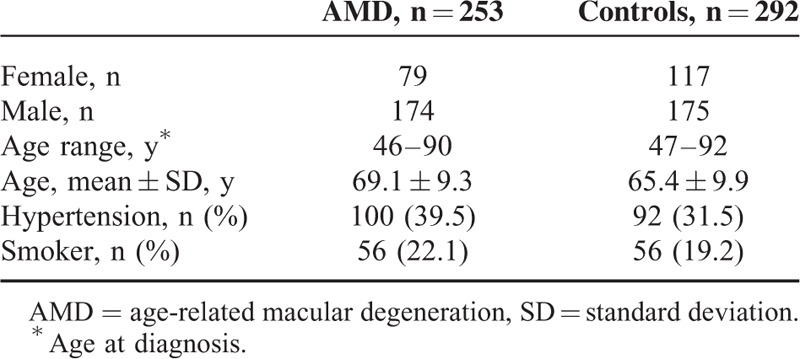
Demographic Distribution of the Study Subjects

In AMD group, 200 patients are affected with neovascular AMD and 53 patients are with atrophic AMD. Among those who diagnosed with neovascular AMD, 134 are with classic CNV and 66 are with occult CNV. The genotyping data of rs12778366, rs3740051, and rs4746720 showed significant deviations from Hardy–Weinberg equilibrium tests in the AMD group but not in the control group (*P* > 0.05, Table 4). The details of the allele, genotype frequencies, and summary statistics for these 3 SNPs are shown in Table 5. None of the codominant, dominant, recessive, or allele model revealed any significant association between rs3740051 and rs4746720 with AMD (*P* > 0.05). However, rs12778366 was found to be significantly associated with AMD in recessive and codominant model (*P* < 0.05). We estimated the OR for homozygous carriers of the risk allele C to be 3.227 (95% CI: 1.015–10.265; *P* = 0.036) for AMD.

**TABLE 4:**
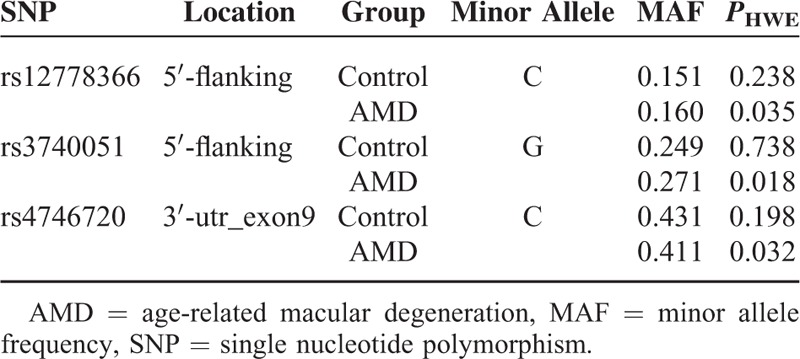
Hardy–Weinberg Equilibrium Test of the Study Subjects

**TABLE 5:**
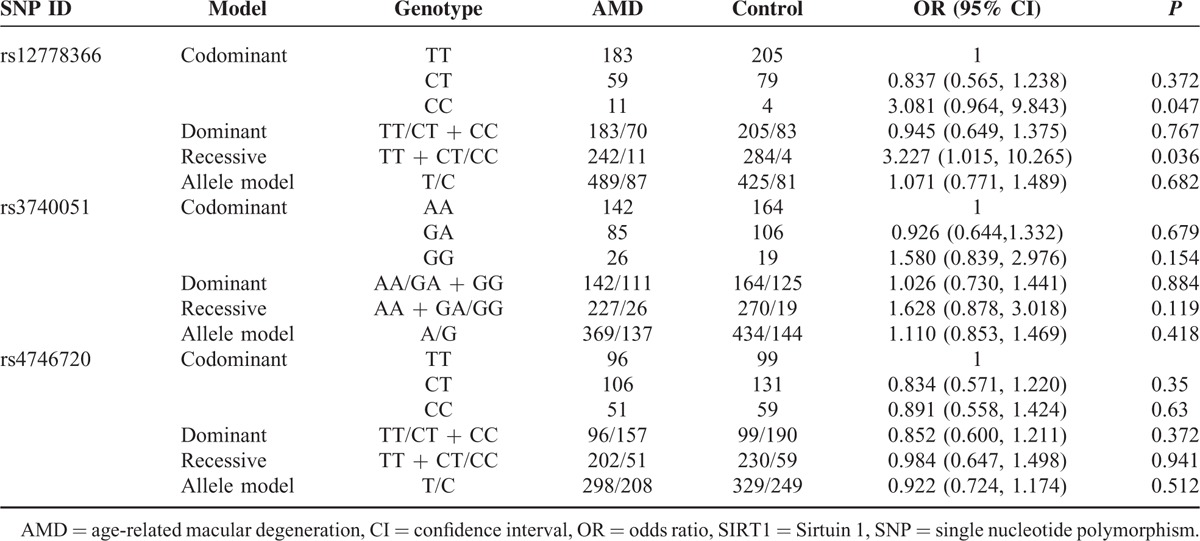
Summary of Genotype Association Analysis on Gene SIRT1

## DISCUSSION

Mutations in SIRT1 gene have been implicated in obesity, diabetes, Parkinson disease, and myocardial infarctions.^12,23,26–28^ After literature reviewed, we selected 3 well-studied SNPs in the SIRT gene, and genotyped a sample of individuals with AMD. rs12778366 and rs3740051 are located at the 5′-flanking region of SIRT1 gene, and rs4746720 located at 3′-UTR of SIRT1 gene. According to the NCBI database, none of these 3 SNPs has definite functional significance. However, some evidences indicated that these 3 SNPs might have potential regulatory function in gene expression.^19,24^ In our control group, the minor allele frequency (MAF) of rs12778366, rs3740051, and rs4746720 are 0.151, 0.249, and 0.431, respectively. In HapMap3 database, the MAF of these 3 SNPs in CHB (Han Chinese in Beijing, China) population are 0.144, 0.311, and 0.4, respectively. We showed that the SIRT1 rs12778366 polymorphism is associated significantly with AMD, and estimated the OR in recessive model for homozygous carriers of rs12778366-C at 3.227 for AMD. We also adjusted this result by gender and age using logistic regression analysis, and got a corrected *P* value of 0.016. Subtype regression analysis also showed significant in both neovascular AMD and atrophy AMD group (*P* = 0.030 and *P* = 0.007, respectively). Though genotyping data showed significant deviations from Hardy–Weinberg equilibrium tests in AMD group, lots of association studies do not require patients group to be in HWE. These studies consider departure from Hardy–Weinberg equilibrium in patients group as a biological consequence rather than genotyping error, and only require control samples to be in HWE.^29–31^

AMD is the leading cause of irreversible central vision loss in elderly Chinese population. Linkage and association studies have implicated genetic modulators of AMD risk related to many mechanistic pathways, including oxidative stress, complement system dysregulation, DNA repair, mitochondrial dysfunction, neovascularization, and microglial recruitment.^32^ Researchers have tried to find genetic determinants for AMD and have proved that SNPs of many genes (eg, CFH,^33^ ARMS2,^34^ ELN,^35^ SKIV2L^36^) associated with AMD.

The expression levels of SIRT1 are different in diverse types of tissues and organs. Jaliffa et al^37^ reported that SIRT1 is localized in the nucleus and cytoplasm of cells in all normal ocular structures. Peng et al^38^ examined SIRT1 expression, found that SIRT1 decreased in human and rats aged eyes, and concluded that this change may be relevant to the self-renewal capacity of retinal stem cells. Anekonda and Adamus reported that resveratrol up-regulates SIRT1 in retinal cells and protects cells from apoptotic death induced by anti-retinal antibody,^39^ while down-regulation of SIRT1 causes retinal damage through multiple mechanisms.^38,40,41^ These results suggest that SIRT1 may play a role in the protection of the retina and optic nerve against degeneration. There are an increasing number of papers assessing the relationship between SIRT1 and AMD in animal and in vitro studies.^17,18,42^ rs12778366 located in the promoter region of SIRT1 gene and has been studied in the schizophrenia, type 2 diabetes, systemic lupus erythematosus, glucose tolerance, and longevity patients.^20,25,43,44^ Lots of studies showed rs12778366 is a possible functional SNP in SIRT1 gene. Sylwia et al reported that carriers of the minor allele of SNP rs12778366 had better glucose tolerance. Recently, Hu et al^45^ implied that rs12778366 CC homozygotes may affect the SIRT1 mRNA expression. These results indicated that rs12778366 in SIRT1 may play a crucial role in gene function. We hypothesize that variants in rs12778366 may account for down-regulation of the protein, since this SNP is located in transcription factor binding site (TFBS).

The major limitation of this study is the relatively small sample size and lack of replication group. Since the molecular mechanism how this genetic mutation influences AMD pathogenesis is not fully understood yet, larger size studies from different population are necessary to confirm the association between this variant with AMD. Furthermore, functional studies of rs12778366 should be conducted for further investigation.

In conclusion, we found that the rs12778366 within the promoter region of SIRT1 was nominally associated with susceptibility to AMD in this Chinese Han population. This finding provides further insight into the underlying genetic character and pathophysiology of the development of AMD.
